# Assaying activity-dependent arteriole and capillary responses in brain slices

**DOI:** 10.1117/1.NPh.9.3.031913

**Published:** 2022-05-04

**Authors:** Danica Bojovic, Teresa L. Stackhouse, Anusha Mishra

**Affiliations:** a Oregon Health & Science University, Jungers Center for Neurosciences Research, Department of Neurology, Portland, Oregon, United States; b Oregon Health & Science University, Vollum Institute, Portland, Oregon, United States; c Oregon Health & Science University, Knight Cardiovascular Institute, Portland, Oregon, United States

**Keywords:** neurovascular coupling, *ex vivo*, acute brain slice, arteriole, capillary

## Abstract

**Significance:**

Neurovascular coupling (NVC) is the process that increases cerebral blood flow in response to neuronal activity. NVC is orchestrated by signaling between neurons, glia, and vascular cells. Elucidating the mechanisms underlying NVC at different vascular segments and in different brain regions is imperative for understanding of brain function and mechanisms of dysfunction.

**Aim:**

Our goal is to describe a protocol for concurrently monitoring stimulation-evoked neuronal activity and resultant vascular responses in acute brain slices.

**Approach:**

We describe a step-by-step protocol that allows the study of endogenous NVC mechanisms engaged by neuronal activity in a controlled, reduced preparation.

**Results:**

This *ex vivo* NVC assay allows researchers to disentangle the mechanisms regulating the contractile responses of different vascular segments in response to neuronal firing independent of flow and pressure mediated effects from connected vessels. It also enables easy pharmacological manipulations in a simplified, reduced system and can be combined with ${\mathrm{Ca}}^{2+}$ imaging or broader electrophysiology techniques to obtain multimodal data during NVC.

**Conclusions:**

The *ex vivo* NVC assay will facilitate investigations of cellular and molecular mechanisms that give rise to NVC and should serve as a valuable complement to *in vivo* imaging methods.

## 1 Introduction

The brain is one of the most energetically costly organs relative to mass, yet it has limited energy reserves.^1^^,^^2^ To ensure proper supply of metabolic substrates to active neurons, blood vessels typically dilate in response to neural activity, thus increasing blood flow to meet the increased energy demand. This phenomenon is termed functional hyperemia and forms the basis of functional neuroimaging techniques. It is driven by multiple parallel signaling processes between neurons and the vasculature, collectively termed neurovascular coupling (NVC).^3^^,^^4^ NVC depends on the cooperation of several cell types that comprise the neurovascular unit: neurons, glial cells, endothelial cells, and vascular mural cells [vascular smooth muscle cells (VSMCs) and pericytes]. Neurons can release vasoactive molecules directly onto blood vessels driving changes in vessel diameter and, therefore, blood flow. Astrocytes can also sense neural activity and consequently release vasoactive substances onto blood vessels (for extensive review, see Refs. 3–6). The vessel response depends on resting conditions of the neurovascular unit and the type of vasoactive molecule(s) released.^7^^–^^10^ The neurovascular unit changes over development and under pathological conditions, and NVC mechanisms also change in concert, underlining the plasticity of these mechanisms.^4^^,^^6^

In this paper, we describe an *ex vivo* approach for studying vascular responses evoked by neuronal activity, i.e., NVC, using acute brain slice preparation. Our goal is to provide an efficient method to perform NVC experiments in a controlled, reduced preparation, which can be optimized by researchers and combined with other *ex vivo* techniques to address their particular research question. While protocols to perform imaging of blood vessels and their associated mural cells have been previously described,^11^^,^^12^ this paper extends those methods to allow the study of endogenous vasoactive mechanisms engaged by neuronal activity. We describe how to concurrently monitor electrically stimulated neuronal activity and the subsequent change in vascular diameter, with emphasis on proper placement of electrodes near the blood vessel to ensure regional neuronal activation without direct vessel stimulation. We discuss these methods in the context of the cerebral cortex, however, similar approaches can be easily adapted for other brain regions such as the hippocampus^13^ and cerebellum.^14^

Activity-dependent NVC experiments in acute brain slices consist of two major components: first, electrically stimulating the brain slice to induce and record neural activity, and second, time series imaging of nearby blood vessels to capture the dynamic neural activation-induced changes in diameter. This technique is valuable as an addition to traditional electrophysiological techniques because it can be combined with whole-cell patch-clamp or field potential recording, as well as ${\mathrm{Ca}}^{2+}$ imaging. Therefore, this method allows researchers to study the interactions between energy homeostasis and neural processing in the brain.

Several factors related to acute brain slice preparations must be considered prior to *ex vivo* NVC experiments to obtain robust and reliable results. Although not an exhaustive list, the primary considerations are discussed in the following sections.

## 2 State of Blood Vessels in *ex vivo* Brain Slices: Re-Establishing Vessel Tone

Once the brain is isolated from the body and sliced, blood vessels are cut open, and they lose the perfusion pressure driven by the circulatory system. Therefore, *ex vivo* brain slice preparations consist of small segments of blood vessels that are disconnected and depressurized, resulting in one of two possibilities: maximally dilated atonic vessels or collapse of the vessel lumen, likely due to pressure from surrounding tissue.^11^ Blood vessels can respond to neural activity by constricting or dilating, but vessels that are maximally dilated will not be able to dilate any further and therefore dilatory signals will be completely masked. The resting tone of vessels can dictate their response to activity,^8^ therefore, further biasing these maximally dilated vessels toward constriction. Therefore, to visualize both dilation and constriction events in an unbiased manner, resting vessel tone must be re-established by incubating the slices with a vasoconstrictive substance at low concentrations.

Our lab and others have successfully used the synthetic thromboxane $A_{2}$ analog U46619 to induce vessel tone.^9^^,^^15^^,^^16^ U46619 activates thromboxane A2 receptor, a $G_{q}$-coupled receptor on VSMCs and pericytes, which activates inositol trisphosphate 3 (${\mathrm{IP}}_{3}$)-dependent increase in ${\mathrm{Ca}}^{2+}$ and causes contraction the mural cells,^17^ subsequently constricting the vessels. Other vasoconstrictors, such as noradrenaline or adenosine triphosphate (ATP) have also been used by ourselves and others.^14^^,^^15^ However, receptors to these molecules are also present on nonvascular cells (neurons, astrocytes, microglia, etc.), where they underlie a multitude of functions. Thus, using noradrenaline or ATP may affect neurons, glia, and vasculature independently, which makes them unsuitable for NVC experiments. In contrast, thromboxane receptors are not expressed in neurons or astrocytes,^18^^–^^21^ thus minimizing unintended effects, and we have gravitated toward using U46619 as our constrictor of choice.^15^^,^^22^ Our lab uses 200 nM U46619, which constricts both arterioles and capillaries by 15% to 20%.^15^^,^^22^ We find that this concentration of U46619 gives the vessel the level of constriction that is comparable to the physiological tone. *In vivo* studies show that the resting diameter of first- to fourth-order capillaries ranges between 3.7 and 5.5  μm, with an average diameter of 4.4  μm, whereas the penetrating arteriole diameter averages ∼12.5  μm.^14^ These values are similar to what we observe in the slice after preconstriction with 200 nM U46619, which gives us confidence that we are in the correct range of resting tone for capillaries (see supplementary data shown in Ref. 22). Furthermore, dilations observed *in vivo* are generally in the 10% to 20% range for both capillaries and arterioles.^14^^,^^23^^,^^24^ This indicates to us that obtaining a preconstricted tone that is 15% to 20% of the baseline diameter in a slice puts the vessel in the range where similar dilations as *in vivo* could be observed without hitting a “ceiling” of the dynamic range while still allowing room for constrictions, which can occur under certain physiological conditions.^25^^,^^26^ In addition to providing resting tone, U46619-evoked constriction is an indication that the blood vessel is sufficiently healthy and responsive to vasoactive signals and serves as a checkpoint for proceeding with the experiment. It is important to avoid collapsed vessels for any functional experiments as it is near impossible to regain dynamic regulation of such vessels.

## 3 Identifying Different Types of Blood Vessels

The vascular tree is composed of several distinct segments—arteries, arterioles, capillary beds, venules, and veins—that differ structurally and functionally. Cerebral arteries emerge from the circle of Willis and spread out around the brain to the pial surface where they branch into pial arteries. Arterioles emerge from these pial arteries and penetrate radially into the cortex where they branch out into the network of capillaries that infiltrates the tissue. The capillaries ultimately join into venules, through which blood is drained back to the pial veins.^27^ Arterioles and capillaries can independently respond to neuronal- and glial-derived vasoactive signals, governed by VSMCs and pericytes, respectively.^15^

**Fig. 1:**
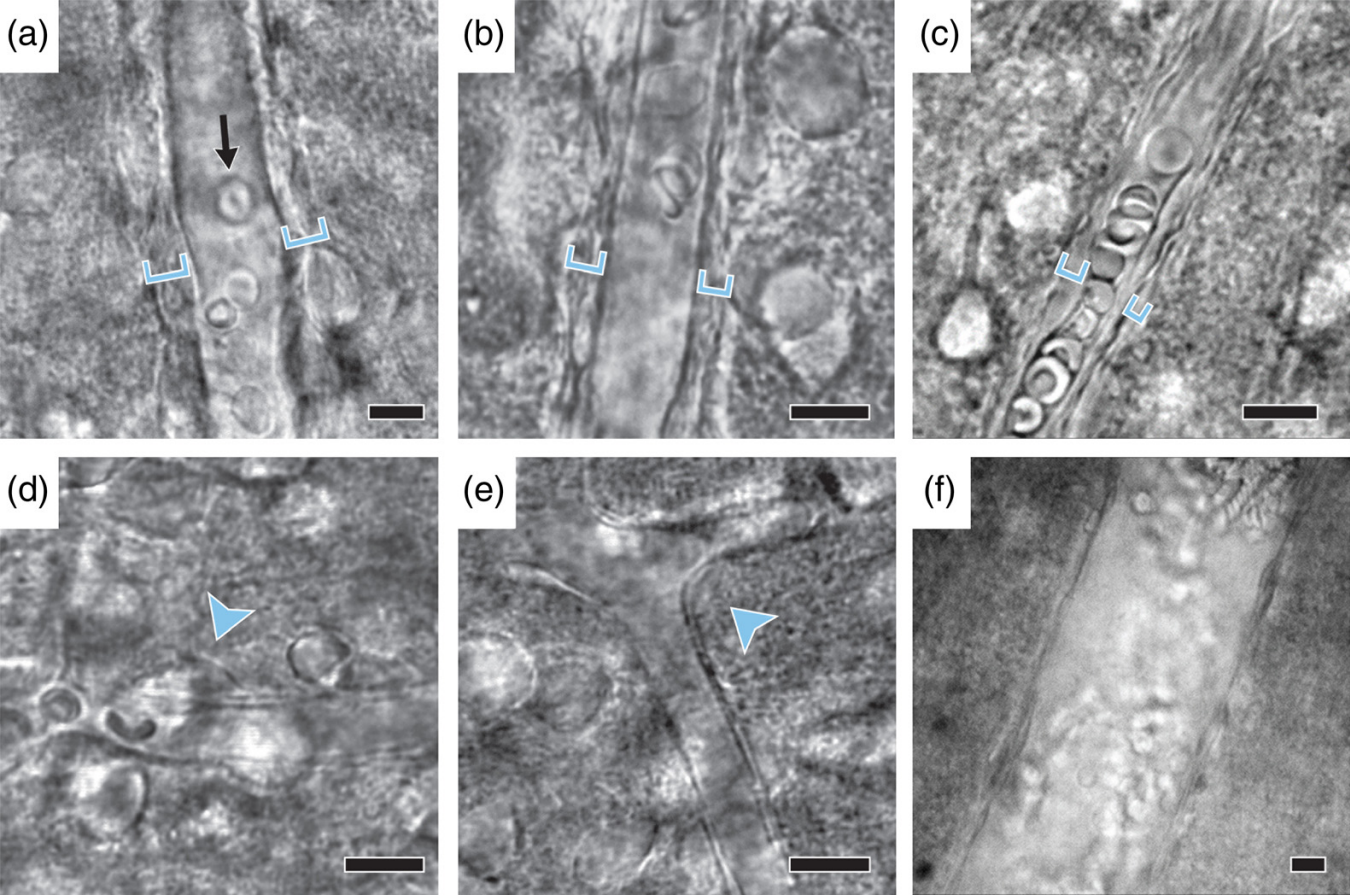
Examples of blood vessels in cortical slices. (a), (b). Examples of arterioles demonstrating the larger vessel diameter and thick abluminal walls (marked with brackets) where the VSMC layer is visible. Note the size of the vessels compared with the red blood cells visible within the lumen. (c). A transition zone between arteriole and capillary depicting the smaller vessel diameter and thinner wall compared with arterioles. Although the VSMC layer is not clearly visible, the abluminal wall is thicker than that expected of capillaries. (d), (e). Example capillaries depicting their narrower lumen and thin endothelial wall. Pericytes can often be observed as individual cell bodies attached to the outer wall (arrowheads). (f) Venules have a large diameter and thin endothelial walls with sparse VSMCs. Scale bars: 10  μm.

Arterioles can be easily recognized by their relatively large vessel diameter (>10  μm) and thick wall consisting of a contiguous layer of abluminal VSMCs [Figs. 1(a) and 1(b)]. Under brightfield or differential interference contrast (DIC) optics, the VSMC rings are sometimes clearly visible, and other times it looks like a thick uneven outer layer of the arteriole. Capillaries have a visibly narrower lumen (∼5 to 8  μm diameter in slices before preconstriction), approximately the size of red blood cells, with a smooth thin endothelial wall and stereotypic pericyte cell body located abluminally with a “bump-on-a-log” morphology^28^^,^^29^ [Figs. 1(d) and 1(e)], often slightly indenting the capillary lumen inward, at ∼50  μm intervals.^14^ Distinct capillary regions are connected with distinct arteriole regions by arteriole-capillary transition zones, which are often slightly wider than capillaries but have fewer and less contiguous VSMCs than arterioles, making them difficult to classify as either capillary or arteriole [Fig. 1(c)]. These transitions zones likely play an important role in CBF regulation and NVC;^30^ however, as they are difficult to definitively identify using brightfield microscopy, we leave them out of this protocol paper. Investigating these segments will require confocal or two-photon imaging modalities combined with genetic reporter lines expressing fluorescent proteins in vascular mural cells. At the other end of the vasculature, venules typically exhibit a larger diameter and a thin endothelial wall with sparse VSMCs [Fig. 1(f)]. Venules are covered by fewer VSMCs that are less contractile; therefore, one telltale sign of having accidentally selected a venule is the lack of constriction in response to U46619. The experiment should be abandoned in such cases. Cerebrovascular regulation differs between vascular segments,^30^ thus it is crucially important to define the criteria for the type of blood vessel of interest before starting the experiment. Depending on the question, more specific identification of vessel segments and mural cells can be made using reporter lines crossed with the NG2-Cre or PDGFRβ-Cre mice for pan mural cell labeling^29^ or the newly identified Atp13a5-Cre line, which specifically labels pericytes in the central nervous system.^31^

## 4 Measuring Field Potentials

A crucial step in NVC experiments is to activate the neuronal tissue surrounding the vessel of interest. To stimulate neurons in acute cortical brain slices, we deliver an electrical stimulation in layers I/II of the cortex, where intracortical and a subset of thalamocortical inputs synapse onto the dendrites of pyramidal neurons. The resulting fiber volley (summated action potentials) and field excitatory postsynaptic potentials (fEPSPs) can be recorded in deeper cortical layers. The stimulating electrode should not be in direct contact with the vessel of interest. If so, VSMCs on the blood vessels can also depolarize and constrict in response to direct stimulation, integrating with the NVC signals and confounding results. The field potentials may also be recorded in voltage clamp mode as the current required to hold the electrode at 0 mV, thus called field excitatory postsynaptic currents (fEPSCs). Depending on the circuitry of some types of patch-clamp apparatus, the voltage-clamp mode can produce recordings with significantly less noise and has been used in some papers.^15^ Note that the waveform of the current change for the fEPSC (when using conventional signage) is inverted from that of the fEPSP.

The position and direction of cortical pyramidal neuron dendrites guide the placement of electrodes in the slice. In the cortex, dendrites of pyramidal neurons project toward the pial surface, ending up roughly perpendicular to the pial surface. Therefore, the dendrites will be projecting in different directions depending on the slice and region of interest (ROI). Electrodes should be placed accordingly to follow the path of the circuitry. It is also important to account for the depth and angle of dendrites in relation to neuronal cell bodies adjacent to the vessel of interest. This is determined by the brain orientation when slicing as well as the area of cortex. Coronal slices from the mid-region of the brain (underneath the bregma) will have neurons with dendrites projecting in the same plane, whereas frontal or caudal regions will contain more dendrites that are slanting outward or inward compared to the slice plane. Therefore, the recording and stimulating electrodes need to be placed in accordance with the direction of dendrites with reference to the slice plane to correctly stimulate the area of interest. If the slope of the dendrites is angled toward the surface of the slice, the stimulating electrode must be placed shallower compared with the recording electrode. In contrast, if the slope of the dendrites is angled deeper within the slice, the stimulating electrode must be placed deeper compared with the recording electrode. Detailed instructions on electrode placement are described in the step-by-step protocol section.

This paper mainly focuses on methods required to measure field potentials to assure that the neural tissue surrounding the blood vessel of interest is activated. We do so by delivering a test pulse of 200 to 300  μA and recording the resultant current in the tissue. It is important at this stage to ensure that the stimulation does not directly stimulate the vessel. During NVC experiments, we deliver a 3 s 20 Hz low frequency stimulation after preconstriction with 200 nM U46619 and monitor both the evoked neural activity and the subsequent vessel response concurrently. This is a simple but gross method to stimulate field activity and the 200 to 300  μA stimulation has worked reliably in our hands. For more precise tuning of the stimulation intensity, a range of test pulses (from low to high intensity) can be administered in relatively quick succession to find the maximal field activity and a pre-set intensity strength (e.g., 50% or 80% of maximal response) can be consistently applied during the NVC assay. We also recommend verifying that the blood vessel is responding to neuronal activation and not to the electrical stimulation itself by performing parallel, interleaved control experiments in the presence of tetrodotoxin (TTX; voltage-gated sodium channel blocker). Preventing neurons from firing with TTX should abolish the blood vessel response (see Ref. 15). Note that this control experiment with TTX does not need to be performed for every experiment, but it is a good way to examine one’s technique in electrode placement and ensure that neuronally driven vascular response (i.e., NVC) is being measured rather than artifactual responses to stimulation.

## 5 Nutrient and Oxygen Supply to Maintain Slice Health

Any experiment performed in *ex vivo* acute brain slices requires particular care to ensure the tissue is healthy and as similar to *in vivo* conditions as possible. This is especially important for NVC experiments as all cellular components—neurons, astrocytes, vascular mural cells, and endothelial cells—over a large ROI need to stay functionally intact and healthy. The blood vessel of interest must be in focus and the lumen visibly open (not collapsed), surrounded by plump, clearly visible neuronal cell bodies (see Ref. 11, for examples, of collapsed and unhealthy vessels). Swollen or shrinking neuronal or other cell bodies indicate poor tissue health, therefore areas with these types of cells should be avoided. The vascular mural cells (VSMCs for arteriole and pericytes for capillaries) are often clearly visible and can be detected while changing focus above and below the plane of the vessel. Endothelial cells are often visible as a thin layer; a swollen endothelium indicates unhealthy tissue and should be avoided. Glial cell bodies are often smaller and more difficult to locate without extensive practice, but we can assume their health if neurons and vascular cells are visibly healthy. All solutions must have an ionic composition that is similar to the cerebrospinal fluid, pH balanced (∼7.4), isotonic for brain tissue (295 to 305 mOsm), and well-gassed to supply adequate oxygen. Below is a rationale for each solution used in different steps of the slice preparation and *ex vivo* NVC assay.

### 5.1 Slicing and Storage Solutions

During dissection and slicing, the brain undergoes major injury, culminating in inflammation and neuronal overexcitation that can be neurotoxic. We use specially designed slicing and storage solutions to minimize neuronal excitability and improve slice health (as described previously in Refs. 11 and 15). After the brain is dissected out of the skull, it is placed in ice-cold “slicing solution” for positioning and slicing. This solution contains N-methyl-D-glucamine (NMDG; neutralized with HCl, instead of ${\mathrm{Na}}^{+}$) to prevent neurotoxicity. NMDG is a positively charged large compound that cannot cross the cell membrane or pass through ion channels, but it sustains the charge gradient across the cell membrane and therefore helps maintain membrane potential. The cold temperature helps slow down biological activity and further reduce overexcitation. After slices are cut, we incubate them in the same NMDG-based slicing solution at a warm temperature (34°C) for 15 to 20 min before transferring slices to a “storage solution,” which is identical to the slicing solution except the NMDG is replaced with ${\mathrm{Na}}^{+}$ to re-establish the ionic gradient across cell membranes. Both slicing and storage solutions contain kynurenic acid to block glutamate receptors and prevent postsynaptic activity. The slicing solution also contains a high concentration of ${\mathrm{Mg}}^{2+}$ to decrease NMDA receptor opening and low ${\mathrm{Ca}}^{2+}$ to minimize presynaptic neurotransmitter release, whereas both are returned to standard concentrations in the storage solution. Finally, both solutions contain ascorbate and pyruvate as antioxidants, which, in our hands, increases the chance of finding healthier blood vessels over the course of the experiments.

### 5.2 Artificial Cerebrospinal Fluid

This solution is used as the bath solution for performing the experiments. It is similar in composition to other recipes used in neuroscience research and contains the basic ionic balance and nutrients to support tissue health. Although the recipe provided below, if followed closely, is designed to produce the necessary pH and osmolarity, we recommend always checking both pH (∼7.4) and osmolarity (295 to 305 mOsm). This is a good practice that may catch errors in solution preparation and can save one a lot of time and valuable tissue.

### 5.3 Bubbling the Tissue with Gases

The *ex vivo* brain tissue depends entirely on the oxygen supplied in the solutions for survival. The brain dissection and slicing procedures are performed in 95% $O_{2}$, 5% ${\mathrm{CO}}_{2}$ bubbled solutions. However, this gas composition is not suitable for performing neurovascular experiments, as high $O_{2}$ levels can alter the function of enzymes that generate vasoactive substances and therefore bias the vessel response toward constrictions.^10^^,^^14^^,^^32^^,^^33^ Therefore, during experiments, we always use the more physiological $O_{2}$ concentration of 20% with 5% ${\mathrm{CO}}_{2}$ (to maintain pH), balanced with 75% $N_{2}$. The level of $O_{2}$ and ${\mathrm{CO}}_{2}$ in the bubbled solutions equilibrates slowly and requires at least 20 min of bubbling to reach equilibrium. It is crucial to keep all the solutions saturated with gases by continuous mild bubbling.

## 6 Why Use 20% $O_{2}$ to Bubble aCSF?

$O_{2}$ itself can induce vasoconstriction at high concentrations, likely due to the effect of $O_{2}$ on enzymes metabolizing arachidonic acid (as discussed in detail in Ref. 33), such that more of the vasoconstrictive 20-HETE is made in the presence of high levels of ambient $O_{2}$. We believe that the tone observed in 20% $O_{2}$ is more physiological. Although many slice experiments (especially electrophysiology) are performed at 95% or 100% $O_{2}$, depending on the type of buffering system used (${\mathrm{CO}}_{2}\text{-}{{\mathrm{HCO}}_{3}}^{-}$ or HEPES), we have not found a good argument to support this practice except convention. $O_{2}$ concentrations have been measured in the brain *in vivo*, where it was reported in the range of 12 to 60 mmHg using Clark-type polarographic microelectrodes by studies performed in the early 2000s,^34^ while more recent studies using two-photon sensitive fluorescent $O_{2}$ probes have reported it to be ∼23  mmHg on average with a range between 5 and 40 mmHg.^35^ In *ex vivo* experiments performed in high $O_{2},$ concentration of $O_{2}$ measured in the tissue was almost ∼16 times higher (>500  mmHg).^32^ In contrast, in 20% $O_{2}$, tissue concentration of $O_{2}$ was measured in the range of 14 to 20 mmHg in both retina^32^ and hippocampus^10^ in the depth range where NVC experiments are performed (determined by the optics of transmitted light on the microscope, usually 20 to 70  μm from the slice surface). These values are much more in line with *in vivo* measurements. Indeed, even in the red blood cell itself, $O_{2}$ concentration was measured to be only ∼60  mmHg.^35^ The first *ex vivo* NVC studies were performed under high $O_{2}$ and surprisingly reported that neural activity led to constrictions (see Ref. 33 for a more complete discussion of the literature). But under the lower $O_{2}$ concentrations (achieved by 20% bubbling), which mimics *in vivo* values, NVC leads to dilations in multiple CNS regions (retina,^32^ hippocampus,^10^ cortex, and cerebellum^14^), as predicted. Thus, we firmly believe that performing *ex vivo* NVC experiments in 20% $O_{2}$ is more physiologically appropriate and imperative for understanding the relevant mechanisms.

It is also important to note, however, that the metabolic demand of brain slices may be higher than *in vivo* and high frequency, sustained neuronal stimulation can lead to run down of synaptic activity in slices.^36^ We expect this is less of a concern for the short 3 s stimulation described in this protocol, where the lower $O_{2}$ concentration minimizes production of vasoconstrictors such as 20-HETE (which mostly occurs only in pathological situations *in vivo*). Lower $O_{2}$ (20%) may not always be the best choice for all studies, especially if NVC evoked by strong or high-frequency activity is the question at hand. Increasing the flow rate may be a useful method to maintain $O_{2}$ replenishment of the tissue and reduce metabolic stress^36^ without altering NVC signals.

## 7 Preparation of Slices Depends on Animal Age

### 7.1 Dissection Speed Matters, But Do Not Rush the Dissection

The brain undergoes quick disruption and cellular death following euthanasia because blood circulation stops and therefore delivery of all metabolic substrates including $O_{2}$ and glucose stops. Therefore, the most damage occurs when brain tissue is “in the dry” while being dissected out of the skull and not yet immersed in bubbled solutions that provide these necessary substrates. Even after the whole brain is dunked in the oxygenated solutions, diffusion constrains continue to expose the inner regions to hypoxia. Therefore, both the speed of dissection and slicing matter. This is one of the main reasons why the tissue looks more damaged in initial stages of learning the slice preparation, as beginners take longer to perform the dissection that should ideally occur within a couple of minutes. The best approach is to go slow at the beginning to gain technical proficiency and precision of dissection, then improve efficiency over time to optimize the slicing technique. Practice will make perfect, eventually.

### 7.2 Handling Young Animal Tissue (P5-P21)

Neural cells in young animals are generally more resistant to hypoxia and will maintain healthy appearance even hours after dissection. However, young animals have a less developed cerebrovascular system,^37^ so it can be more challenging to find healthy blood vessels. Therefore, it is beneficial to cut as many slices as possible when working with young animals to increase the chances of finding blood vessels in the desired brain region. Brain slices from P5-P21 range have been used most widely in neuroscience research as the slices can be readily maintained *ex vivo*, and the basic circuitry is starting to be established; however, it is still a developing brain undergoing rapid changes in neuron and astrocyte number, structure, and connectivity, not to mention vascular development. This also means that a larger variability may exist in their NVC responses. These factors should be considered in the interpretation of data obtained. Working with animals younger than P5 may require a special method of euthanasia, as dictated by the Association of Veterinary Medical Association and approved by the local IACUC. As they have very little microvasculature, we do not use them nor discuss them here.

### 7.3 Handling Young Adult and Adult Tissue (>P21)

As the animals mature, especially rats, their skulls grow thicker and significantly more connective tissue develops, so brain dissection can be more challenging. A spring loaded Rongeurs tool is ideal for cutting the skull of adult rats, but must be done carefully to avoid shearing the brain tissue. In these animals, cutting along the sagittal suture has a high likelihood of damaging the cortical tissue, so it is preferable to cut laterally on both sides of the skull and remove the skull in one piece without damaging the top layer of the cortex. Making a cut in the frontal bone at its narrowest region anterior to the bregma, across the orbits, can help with this step. The circulatory system is established and more robust in adults, making it easier to find and identify intact blood vessels at different levels of the vascular tree. Further, the neuronal-glial network is more mature, which allows the study of fully developed NVC pathways. However, extra care must be taken as brain slices from older animals are less resilient under slicing conditions and tend to deteriorate faster. In our experience, the use of the protective slicing and storage solutions and the warm recovery appear to especially help in obtaining good slices from adult animals that stay healthier for longer.

Tissue integrity may also be affected by the slicer (microtome) employed. The Leica Vibratome has been used broadly in the neuroscience field for slice preparation. In our experience, the vibration principle used by this machine, especially in the z axis, can cause damage to cells that are close to the surface of the slices. This is very well tolerated by young brains, which are generally more resistant to damage, and the lack of significant myelin allows the experimenter to image deeper in the tissue that stays intact. In slices from older brains, however, increased myelination prevents imaging of deeper tissue, and the damage to regions close to the slice surface is irreversible. We have found better success with the Compresstome VT-300 to produce healthy slices from animals of all ages. The Compresstome avoids vibration, as the tissue is embedded in agarose and cut using a slight compression force. Similar results are also reported when using the Vibrocheck function in the Vibratome. Furthermore, the Compresstome yields slices faster, allowing the tissue to be exposed to oxygenated solutions sooner and therefore leading to better overall tissue health. Ultimately, of course, which machine is used depends on what is available. We recommend taking some time to optimize the settings on control tissue of comparable age before performing experiments.

## 8 Criteria for Performing Experiment and Analysis

### 8.1 Before Starting the Experiment

After the brain slices are removed from the storage solution and bathed with artificial cerebrospinal fluid (aCSF), the tissue has a limited life-span and this is especially acute for slices from older animals. Chances of finding a healthy blood vessel decrease with increased time since slicing as well as the longer a slice sits in the aCSF bath. Once a vessel has been found, the NVC assay, as per our protocol, requires at least 20 min to complete. Therefore, it is important to scan through the brain slice in an organized and quick manner to identify vessels of interest as soon as possible. We generally start at the medial edge of the cortex, near the corpus callosum, and systematically scan the slice from the pial surface to deeper layers and back again while focusing up and down, slowly rotating along the cortical arc. It is easy to doggedly search for a blood vessel in the slice, especially when the rest of the tissue looks good, but it is important to find a balance between a hopeful search and moving on to the next slice to maximize your success rate across all available slices. We recommend spending no longer than 10 to 15 min looking for a healthy blood vessel in each slice. If a vessel type of interest that is in focus over a reasonable length (at least 20 to 30  μm long) is not found, then move on to the next slice. Once a vessel is located, evaluate whether to perform NVC experiment by asking yourself several questions:

1. Do I see the features of the healthy blood vessel? You should be able to see the thin endothelial wall and a clearly visible lumen, as well as outer wall and cells around the vessel (pericytes or VSMCs). The endothelial tube should not look swollen and the lumen must not be collapsed.^11^
2. Can I identify healthy-looking neurons visible around the blood vessel? As the goal of the experiment is to activate NVC, the tissue surrounding the vessel must look healthy. This is most easily assessed by checking whether neurons look plump and healthy, rather than swollen or shrunken.
3. Can I place the recording and stimulating electrodes appropriately to record neuronal field potentials? You should be able to identify the direction and plane of the neuronal dendrites in the ROI so as to know where to place your electrodes. Sometimes, the angular orientation of the dendrites with reference to the slice plane may be too steep to be able to place the stimulating electrode far enough away from the recording electrode. Sometimes a great blood vessel might be sitting right next to a harp string that blocks proper electrode placement. While it is sometimes worth troubleshooting this issue (e.g., carefully edging the electrode below the string), other times it may be better to move on to a different slice.
4. Can I measure electrically stimulated field potentials? It is crucial to evaluate electrode placement by delivering a single stimulation pulse (test pulse) and checking for a robust field potential. It is important also to determine the stimulation strength to obtain a preset range of desired field potential amplitude before continuing with the experiment.

### 8.2 Analysis Criteria

The purpose of *ex vivo* NVC experiments is to study whether and how a certain brain process has an effect on blood vessel diameter. It is necessary to measure the diameter of the vessel in all images of the time-stack spanning the experiment, during baseline and following the various manipulations. Therefore, maintenance of focus on the blood vessel at the same depth is critical. Even slight changes in focus can alter the size of the lumen, as moving up and down a longitudinal section of a cylindrical structure is bound to do. It is also necessary that the blood vessel constricts following the U46619 application to assure that the vessel is responsive and has sufficient tone to respond to neuronal activity. Finally, field potentials of sufficient strength need to be evoked during the experiment. Sometimes, the electrodes may shift slightly during the first ∼10 to 15 min of imaging prior to stimulation (e.g., during baseline recording and exposure to U46619 and other drugs of interest) such that the field potential, although tested at the start of the experiment, is no longer present or has lost its strength below the minimum predetermined requirement. These experiments should be excluded from analysis. Thus, the three primary criteria we use to proceed with analysis are: (1) the vessel must stay in focus, (2) the vessel must respond to U46619, and (3) field potential amplitude must pass the predetermined range during the experiment.

## 9 Materials

### 9.1 Drugs and Reagents

• ddH2O
• NaCl (Fisher Chemical, cat. # S271-3)
• ${\mathrm{NaHCO}}_{3}$ (Fisher Chemical, cat. # S233-3)
• ${\mathrm{NaH}}_{2}{\mathrm{PO}}_{4}$ (Fisher Chemical, cat. # S369-500)
• KCl (Fisher BioReagents, cat. # BP366-500)
• ${\mathrm{MgCl}}_{2}$ (Invitrogen, cat. # AM9530G)
• ${\mathrm{CaCl}}_{2}$ (G-BioSciences, cat. # R040)
• D-Glucose (Fisher Chemical, cat. #D16-1)
• NMDG (MP Biomedicals, cat. # 191506)
• L-ascorbic acid (Sigma-Aldrich, cat. # 255564-100G)
• Sodium pyruvate (Acros Organics, cat. # 132150250)
• Kynurenic acid (Sigma-Aldrich, cat. # K3375-5G)
• HEPES (Fisher Bioreagents, cat. # BP310-1)
• Concentrated HCl (Fisher Chemical, cat. # A144-500)

**Caution**: HCl is a strong acid and extremely corrosive; use appropriate PPE (gloves, lab coat, eye-protection) and handle with care.

• NaOH (Fisher Bioreagents, cat. # BP359-500)

**Caution**: NaOH is a strong base and extremely caustic; use appropriate PPE (gloves, lab coat, eye-protection) and handle with care.

• Agarose (Sigma-Aldrich, cat. # A9793-50G)
• U-46619 (Cayman Chemical Company, cat. # 16450)
• Dimethyl sulfoxide (Sigma-Aldrich, cat. # D8418-250ML)
• Isoflurane (anesthetic, e.g., Piramal Critical Care, NDC: 66794-017-10)

### 9.2 Equipment and Supplies

#### 9.2.1 Solution preparation

• Gases for bubbling: 20% $O_{2}/5\%$ ${\mathrm{CO}}_{2}$, balance $N_{2}$ for experiments and 95% $O_{2}/5\%$ ${\mathrm{CO}}_{2}$ for dissection.

**Caution**: Pressurized gas cylinders are heavy and dangerous if they fall. Handle with care. Always keep secured to a wall-mounted cylinder restraint. When moving cylinders, always have the safety cap screwed on well and use a dolly with chain restraints.

• pH meter (e.g., Thermo Fisher Scientific, Orion Star A111)
• Osmometer (e.g., Knauer, K-7400S)
• Heat plate stirrer
• Analytical balance (laboratory scale)
• 1 L glass bottles
• 250 mL plastic nalgene bottles (for freezer storage)
• General lab items: beakers, graduated cylinders (various sizes), spoons and spatulas, magnetic stir, plastic plates for measuring compounds, etc.

#### 9.2.2 Euthanasia

• Anesthesia chamber
• Large, sharp scissors or guillotine (e.g., Braintree Scientific Nemi Guillotine)
• Carcass bag

#### 9.2.3 Dissection

**Fig. 2:**
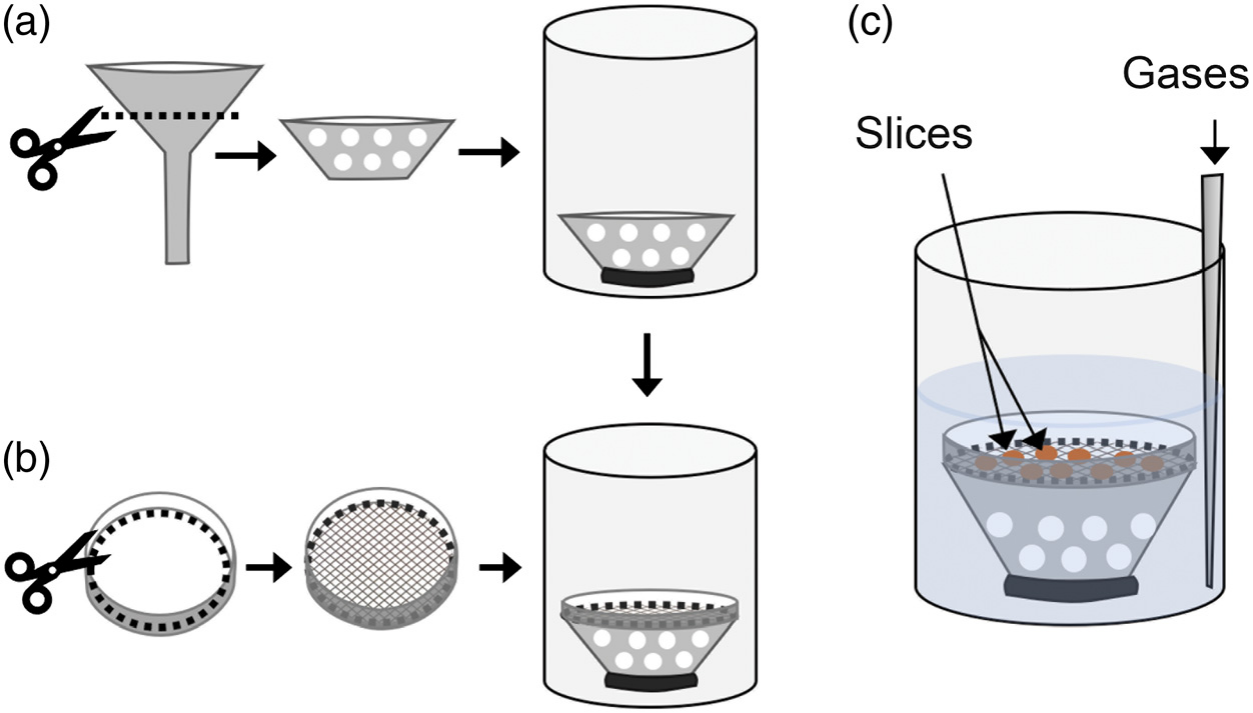
Construction of custom slice storage chambers. These custom handmade chambers contain a raised nylon mesh platform on which the slices sit, thus exposing the tissue to gassed solutions evenly on both sides. These can be easily prepared with a beaker or similar container, a plastic funnel, and a plastic petri dish. (a) Select a funnel that is narrower than the beaker by about 1 cm. Cut the top two-thirds of the funnel away from the stem and punch several holes into the wall of this segment. Attach it to the bottom of the beaker using hot glue. (b) Select a Petri dish that is roughly the same circumference as the funnel mouth. Cut the bottom of the Petri dish away (this can be accomplished quite smoothly using a scalpel blade heated over a Bunsen burner) and glue a nylon mesh over the opening. Carefully glue this dish (nylon mesh facing down) to the mouth of the funnel and let it dry completely. (c) Place the slices on the nylon mesh and a bubbling tube or needle on the side of the chamber. We use a short version of a similar chamber for the warm recovery step so as to use less solution.

• Ice bath for cooling slicing solution
• Tissue slicer such as the Compresstome VF-300-0Z Microtome (Precisionary Instruments Inc.) or Vibratome VT1200 S (Leica Biosystems)
• Warm water bath (e.g., All Stainless-Steel Water Bath Model 181, Precision Scientific)
• Various scissors (e.g., iris scissors, fine scissors)
• Forceps (curved forceps with fine tip and blunt forceps)
• Spatulas (one flat and one angled end)
• Rongeurs bone cutters for adult animals (e.g., #14091, World Precision Instruments)
• Slice transfer pipette with large tip opening (DIY: we use plastic 7 mL Pasteur pipettes with the tip cut off to create an opening roughly the size of the brain slices)
• Petri dish for dissecting brain (we use Sylgard-coated petri dishes, which makes the bottom of the dish less slippery and improves handling of the tissue)
• Cyanoacrylate glue
• Small plastic or glass beaker
• Razor blade
• Slice recovery and storage chambers (see Fig. 2 for DIY instructions)

#### 9.2.4 Imaging

• Vibration isolation table (e.g., Newport Optical table)
• Faraday cage that fits on the isolation table and surrounds the microscope
• Microscope (e.g., Olympus BX51WI) with DIC optics
• Low magnification air objective (e.g., UPlanFL N 4×/0.13, Olympus) and high magnification water-immersion objective (e.g., LUMPlanFL N 40×/0.80  W, Olympus)
• Bright-field light source (e.g., Olympus TH4-100)
• Epifluorescence light source (e.g., EXFO, X-Cite Series 120)
• Camera (e.g., Thor Labs USB 3.0 CMOS Camera or a DAGE-MTI IR-1000 that is more sensitive for IR-DIC imaging)
• Patch-clamp amplifier (e.g., Axopatch 200B, Axon Instruments)
• Digitizer (e.g., Digidata 1450-B, Axon Instruments)
• Isolated current stimulator (e.g., DS3, Digitimer)
• Micromanipulators (e.g., MP-225, Sutter Instrument Company)
• Glass pipette puller (e.g., P-97 Flaming/Brown type micropipette puller, Sutter Instruments Company)
• Imaging chamber (e.g., PC-H, Siskiyou Corporation)
• Thick-walled borosilicate glass capillaries (1B150F-4, World Precision Instruments)
• Thin-walled borosilicate glass capillaries (TW150-4, World Precision Instruments)
• Slice anchor, or a “harp”: this can be created by the user for a completely custom anchor (we make ours using flattened horse-shoe-shaped platinum wire and attaching nylon strings to it) or purchased from commercial retailers (e.g., Warner Instruments)
• Perfusion system (we use a gravity fed system, but perfusion pumps can be employed; make sure the pump is outside the Faraday cage as it can introduce noise into electrical recordings)
• Temperature probe and regulator (e.g., TC-324B, Warner Instrument Corporation)
• Vacuum system plumbed to the imaging chamber

#### 9.2.5 Software

• Image acquisition and analysis software (e.g., μManager ImageJ, NIH, or MetaMorph, Molecular Devices)
• Electrophysiology recording and analysis software (e.g., pClamp Clampex and Clampfit modules, Molecular Devices)

## 10 Reagent Setup

### 10.1 Slicing Solution

Combine 2.5 mM KCl, 1.2 mM ${\mathrm{NaH}}_{2}{\mathrm{PO}}_{4}$, 30 mM ${\mathrm{NaHCO}}_{3}$, 20 mM HEPES, 25 mM D-glucose, 1 mM kynurenic acid, 5 mM Na ascorbate, 3 mM Na pyruvate, 93 mM NMDG, and 93 mM HCl in ∼950  mL of ${\mathrm{ddH}}_{2}O$ (pH 7.3 to 7.4). **Important note:** NMDG is used to replace ${\mathrm{Na}}^{+}$ ions to prevent neuronal depolarization and subsequent overexcitation. NMDG is purchased as a base and must be neutralized with equimolar HCl. ${\mathrm{CaCl}}_{2}$ and ${\mathrm{MgCl}}_{2}$ can precipitate in basic environment, so these compounds must be added after pH adjustment. Stir the solution well to complete the NMDG neutralization step. Next, pH the solution to 7.4, and then add 0.5 mM ${\mathrm{CaCl}}_{2}$ and 10 mM ${\mathrm{MgCl}}_{2}$. Bring the solution to 1 L volume by adding the needed balance of ${\mathrm{ddH}}_{2}O$ using a volumetric 1-L flask. The pH of the slicing solution is balanced by both the ${\mathrm{NaHCO}}_{3}\text{-}{\mathrm{CO}}_{2}$ equilibrium (by bubbling with 95% $O_{2}/5\%$ ${\mathrm{CO}}_{2}$) and HEPES. The addition of HEPES ensures that the solution stays at a neutral pH despite changes in gas solubility at cold (during slicing) or warm (during recovery) temperatures. Measure osmolarity of the solution last; it should be in the 295 to 305 mOsm range. Small deviations can be adjusted with the addition of ${\mathrm{ddH}}_{2}0$ (if solution is hyperosmotic) or NaCl (if solution is hypoosmotic). If the osmolarity differs from this range by more than 10 mOsm, it indicates that something may not have been added in the proper amount and it is best to start a fresh solution. Fresh slicing solution is always best, but it can be aliquoted in 250 ml bottles and stored in −20°C freezer for up to 2 weeks. Thaw completely and stir well before use. **Important note:** Completely thawed slicing solution should be placed on ice and bubbled for at least 20 min to equilibrate $O_{2}$ and ${\mathrm{CO}}_{2}$ before dissection.

As solubility is strongly affected by temperature, solutions that are not fully thawed may have varying solute balance in the icy and melted portions. Therefore, it is important to completely thaw the solution to ensure the solutes are evenly distributed, and then reice while bubbling to make a slushy.

### 10.2 Storage Solution

Combine 92 mM NaCl, 2.5 mM KCl, 1.2 mM ${\mathrm{NaH}}_{2}{\mathrm{PO}}_{4}$, 30 mM ${\mathrm{NaHCO}}_{3}$, 20 mM HEPES, 25 mM D-glucose, 1 mM kynurenic acid, 5 mM Na ascorbate acid, 3 mM Na pyruvate in ∼950  mL of ${\mathrm{ddH}}_{2}O$. Balance pH to 7.4 before adding 2 mM ${\mathrm{CaCl}}_{2}$ and 1 mM ${\mathrm{MgCl}}_{2}$. Bring the solution to 1 L volume by adding the needed balance of ${\mathrm{ddH}}_{2}O$ using a volumetric 1-L flask and mix thoroughly. Measure osmolarity to ensure it is in the range of 295 to 305 mOsm and adjust small deviations as needed. It is best to make storage solution fresh, but it can be stored in −20°C freezer in 250 mL aliquots for up to two weeks. Thaw completely and stir well before use.

### 10.3 aCSF

Combine 124 mM NaCl, 1 mM ${\mathrm{NaH}}_{2}{\mathrm{PO}}_{4}$, 2.5 mM KCl, 26 mM ${\mathrm{NaHCO}}_{3}$, 10 mM glucose, and 1 mM Na ascorbate in ~950  mL of dd $H_{2}O$. Bubble the solution well with 20% $O_{2}/5\%$ ${\mathrm{CO}}_{2}/75\%$ $N_{2}$ for ∼20  min to equilibrate gases and balance the pH. If necessary, adjust the pH to ∼7.4, then add 1 mM ${\mathrm{MgCl}}_{2}$ and 2 mM ${\mathrm{CaCl}}_{2}$. Bring the solution to 1 L volume by adding the needed balance of ${\mathrm{ddH}}_{2}O$ using a volumetric 1-L flask. Measure osmolarity to ensure it is within 295 to 305 mOsm range. **Important note**: For neurovascular experiments, bubble the aCSF with gas containing 20% $O_{2}/5\%$ ${\mathrm{CO}}_{2}$ balanced with $N_{2}$, as high $O_{2}$ levels can bias NVC signaling pathways toward constriction.^10^^,^^14^^,^^32^^,^^33^ **Important note**: aCSF should be freshly prepared to maintain antioxidative properties of ascorbate. If necessary, it can be stored in the fridge but we do not recommend storing it for more than 1 to 2 days. If desired, ascorbate can be omitted and the solution stored for up to 1 week in the fridge. Add ascorbate just before use and mix thoroughly. The glucose content of the aCSF increases the chance of bacterial or fungal contamination. Always check the solution for any visible growth/contamination if storing for more than a day.

**Tip**: Preparing higher concentration stock solutions of each reagent can speed the solution preparation. Consider the solubility of each substance when preparing stock solutions. We typically prepare stock solutions as follows: 4 M NaCl, 1 M ${\mathrm{NaHCO}}_{3}$, 1 M ${\mathrm{NaH}}_{2}{\mathrm{PO}}_{4}$, 1 M KCl, 1 M ${\mathrm{CaCl}}_{2}$, and 1 M ${\mathrm{MgCl}}_{2}$. These stock solutions can be stored in the fridge for up to 2 months. HEPES, D-glucose, Na ascorbate, Na pyruvate, thiourea, kynurenic acid, and NMDG are always added fresh.

## 11 Equipment Setup

### 11.1 Solutions

All solutions should be bubbled for at least 20 min before use. Fill the warm recovery chamber and storage chamber with slicing and storage solutions, respectively. Chambers should be filled such that slices are under at least ∼1  cm of solution. Gases will be continuously leaving the solution at the interface between the solution and room air. Continuous mild bubbling of the solution is needed to maintain correct $O_{2}$ concentration and pH (buffered by bicarbonate-${\mathrm{CO}}_{2}$ equilibrium). Having plenty of solution above the slice net ensures that the gas concentration at the slice is optimal. The warm recovery chamber should be kept in a water bath at 34°C to 35°C and the storage chamber left at room temperature (∼20°C to 22°C). The remaining slicing and storage solutions should be stored on ice. All solutions should be bubbled continuously unless otherwise indicated.

### 11.2 Euthanasia Station

Use an appropriate protocol approved by the local IACUC or the equivalent animal welfare organization. In the case of performing isoflurane anesthesia with decapitation, place the instrument for euthanasia (guillotine for rats or large scissors for mice) immediately adjacent to the isoflurane chamber. Fill a small beaker with prebubbled, slushy slicing solution and place it next to the station (do this step last, so solution remains slushy and freshly bubbled at the time of euthanasia). Depending on the volume of the warm recovery slice chamber, there may not be enough slicing solution for this step. This step is meant primarily to immediately cool the head following decapitation but the brain does not come directly into contact with the solution. Therefore, bubbled storage solution slushy may be substituted if necessary.

### 11.3 Dissection Station

**Fig. 3:**
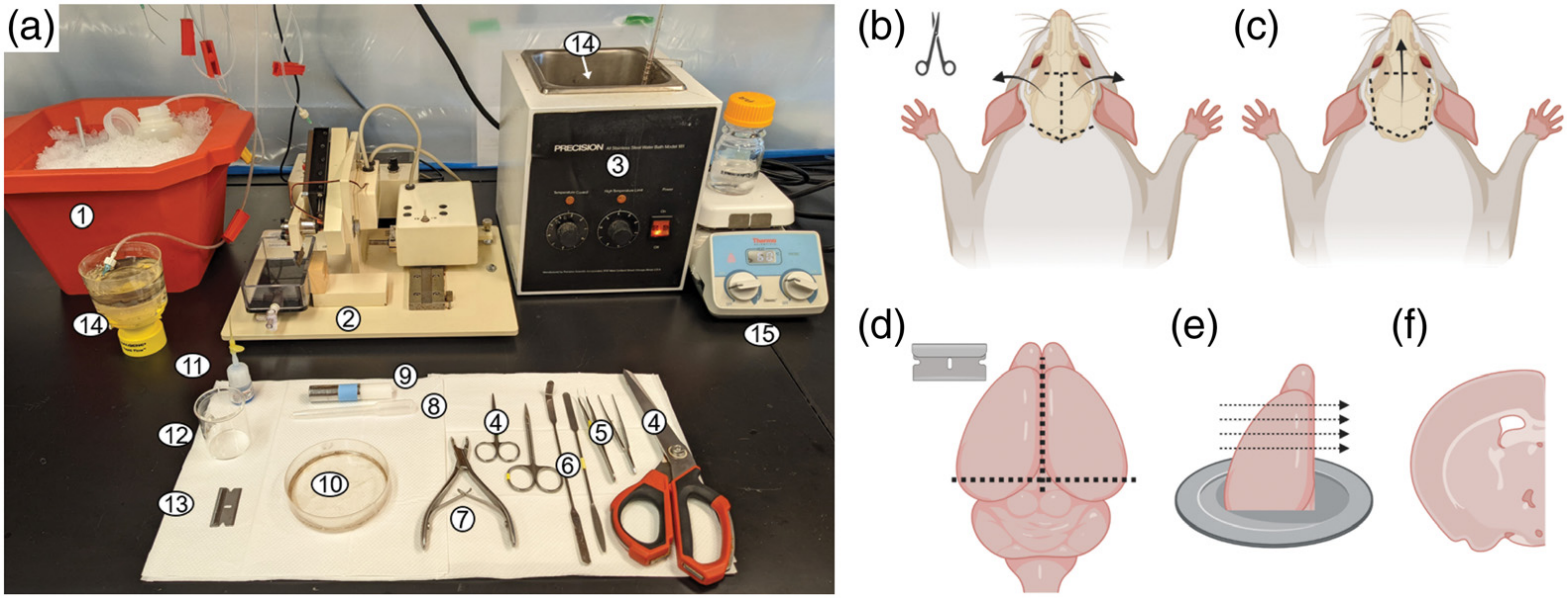
Dissection tools and steps for obtaining cortical brain slices. (a) Dissection tool setup. (1) Ice bath, (2) tissue slicer (Precisionary Compresstome VF-300-0Z shown), (3) warm water bath, (4) various scissors (large scissors, iris scissors, and fine scissors), (5) forceps (curved forceps with fine tip and blunt forceps), (6) spatulas (one flat and one angled end), (7) Rongeurs tool, (8) slice transfer pipette, (9) specimen holder, (10) Sylgard-coated Petri dish, (11) cyanoacrylate glue, (12) small beaker, (13) razor blade, (14) slice recovery (inside warm bath, not visible) and storage chambers (at room temperature), (15) warmed magnetic stir plate for keeping agar solution molten. (b), (c). After cutting the skin with a pair of sharp scissors to expose the skull, cut the skull bone with scissors or Rongeurs tool (depending on skull thickness) to expose the brain. (b) In mice and young rats, the skull bone is cut along the midline and on each side from the base of the neck, then the bones pulled back laterally to expose the brain. (c) In adult rats, it is better to cut the skull laterally and at the bottom on each side and remove it in one piece from the top to avoid damaging the cortex. (d) Using a razor blade, cut away the bottom of the brain (cerebellum plus a small segment of the occipital lobes) and separate the hemispheres along the sagittal midline. (e) Attach one hemisphere upright on the specimen block using cyanoacrylate glue. Arrows indicate slicing direction for obtaining coronal slices. (f) Cut brain slices to appropriate thickness, for example, 300  μM. (b)–(f) Created with BioRender.com.

Lay out dissection tools next to the microtome: Sylgard-coated Petri dish, one straight and one angled-end spatula, skin scissors, skull scissors (for mice and young rats) or Rongeurs tool (for adult rats), blunt tip straight and curved forceps, razor, cyanoacrylate glue, and cooled specimen platform or tube (piece to which the brain is glued for cutting slices). Prepare a clean dissection surface by layering a few paper towels next to the tools [Fig. 3(a)].

### 11.4 Imaging Rig

Make sure all solutions are bubbling with 20% $O_{2}/5\%$ ${\mathrm{CO}}_{2}$ (balance $N_{2}$). Prime the perfusion lines with the control aCSF and make sure it is flowing through the imaging chamber at a rate of ∼3 to 4  mL/min. Turn on the temperature regulator (such as TC-324B) and ensure than solution entering the chamber is warmed to 34°C to 35°C. Start the vacuum line on the other side of the chamber and regulate the outflow making sure that chamber fills sufficiently to fully submerge a slice stably but does not overflow. Turn on the imaging and recording equipment, such as amplifier, digitizer, manipulators, stimulator, light sources, etc. Start the imaging and recording software and preset settings to autosave data files, if necessary and possible (e.g., both pClamp and μManager require file names and folder location to be preset for automatic data storage).

**Tip**: Prior to dissection and slicing, prepare all the solutions you need for the experiments and get them bubbling. We routinely prepare control aCSF and aCSF containing the preconstrictor agent (200 nM U46619). Prepare any other drugs of interest (for example, blockers of putative vasoactive pathways) ensuring that they all contain U46619 at the same concentration. We prepare a large volume of 200 nM U46619 in aCSF and then divide this solution to add other drugs of interest. This ensures that all the experimental solutions have the same concentration of U46619.

## 12 Procedure

### 12.1 Dissection and Slicing

1. Fill the anesthesia chamber inside a chemical fume hood with isoflurane by placing a tissue paper soaked in ∼1  mL of isoflurane inside the chamber and letting it vaporize for ∼2  min (or use an isoflurane vaporizer set to 5%). Place the animal in the anesthesia chamber taking care to ensure that the animal does not come directly into contact with liquid isoflurane (we place the isoflurane-soaked tissue paper in a porous container within the chamber so the animal cannot touch the tissue). After the animal becomes unconscious, observe the chest cavity movement of the animal to monitor breathing. After breathing becomes shallow and slows down considerably (once every 5 to 10 s), wait at least 30 more seconds to make sure the animal is deeply anesthetized.

**Tip**: Younger rodents may stop breathing altogether, but especially in older animals, breathing does not completely stop under isoflurane. Use immobility and shallow breaths at a slow rate to confirm full anesthesia.

2. Remove the animal from the chamber and ensure a lack of responsiveness to a tail or paw pinch to confirm anesthesia depth. Quickly decapitate with large, sharp scissors (for mice and young rats) or guillotine (for adult rats) and immerse the head immediately in the beaker containing bubbled slushy slicing solution.

**Tip**: Ensure euthanasia scissors or guillotine are sharp and appropriately sized for the animal. Perform decapitation in a single snip to minimize suffering for the animal as well as distress for the experimenter.

3. Transfer the head to the dissection station. Place the head on the paper towels and cut the scalp along the midline from posterior to anterior end with a pair of sharp scissors. Fold the skin flaps away laterally to either side to expose the skull [Fig. 3(b)]. It may be necessary to scrape away connective tissue and some muscle (especially in older animals) to expose the skull.

**Tip**: Adult rats have significantly more connective tissue compared with mice and young rats.

4. Dip the head in the beaker of slushy or ice-cold slicing solution every ∼5 to 10 s to keep the brain cool.
5. Cut the skull posterior to anterior along the sagittal suture, followed by two mediolateral cuts on either side of the base of the head to separate the skull bones and surrounding muscle from the neck region [Fig. 3(b)]. Make two further small cuts at the nasal end, to cut the frontal bone adjacent to the ocular orbits. Remove the skull flaps by pulling them upward and out laterally with blunt forceps (straight or curved, depending on experimenter’s comfort) to expose the brain.

**Tip**: Keep the tips of the scissor tilted upward and away from the brain while cutting to avoid damaging the cortex.

**Tip**: Adult rats have thick skulls, so bone cutters (Rongeurs tool) are necessary. Brain damage is more likely because more force is required for cutting, and the cutting edge of bone cutters is much thicker. It is recommended that adult rat skulls are cut laterally on both sides and the top section of the skull removed in one piece to minimize damage to the cortex [Fig. 3(c)].

6. Fill the Sylgard-coated Petri dish (about three quarters full) with freshly bubbled slushy slicing solution. Carefully insert the flat spatula under the brain at the anterior end (frontal lobe) and lift the brain off the skull, dislodging it from the skull and cutting away the optic and cranial nerves under the ventral surface. Quickly remove the whole brain into the Sylgard-coated Petri dish to immerse in slicing solution.

**Tip**: Quick brain dissection is a key determinant of slice quality and therefore vessel and neural health. These steps will take time in the beginning, but with practice, they should become faster. Ensure that the brain spends as little time as possible out of bubbled solution to minimize damage.

7. Position the brain with the dorsal surface facing up in the Sylgard-coated Petri dish. With a razor blade, remove the cerebellum and posterior fifth of the hemispheres with a single smooth coronal cut [Fig. 3(d)]. Use a pair of blunt forceps or a spatula to stabilize the front end of the brain if necessary, without actually squeezing or grabbing the brain tissue.

**Tip**: Cutting the posterior segment of the brain away with the cerebellum creates a flat surface, which will help mount the brain stably on the specimen platform.

**Tip**: Too much solution in the Petri dish can cause the brain to float and slip away from the razor, making it difficult to handle and resulting in unintentional angular cuts. This can be solved by pipetting away some solution so the brain does not float. But take care as too little solution will deprive the tissue of oxygen and may even dry it out, resulting in poor slice quality. Keep enough solution to just cover the brain.

8. With a mid-sagittal cut, split the two hemispheres along the midline [Fig. 3(d)].
9. Prepare the specimen platform by placing a tiny drop of cyanoacrylate glue at its center. Use the angled spatula to carefully lift off one hemisphere of the brain and carefully mount it on the glue with the posterior cut edge facing the specimen platform [Fig. 3(e)].

**Tip**: Too little glue can cause the brain to separate from the specimen platform at later steps, whereas too much glue can wick up the sides and inner cavities of the brain and prevent slices from separating easily (or even damaging the tissue).

**Tip**: When cutting slices on the Vibratome, gluing an agar block behind the brain can provide support and help obtain smoother, even slices (see Ref. 11 for more detailed instructions). The Compresstome slicer requires the brain to be embedded in low melting point agarose to hold the tissue in place on the slice holder. The more closely the agarose density matches tissue density, the smoother the slice cutting will be. We use 2% agarose to obtain cortical slices as recommended by the manufacturer and this has worked well in our hands (see manufacturer website for detailed directions on agarose mounting). When using the Compresstome slicer, extra care should be taken to minimize the amount of glue applied to attach the brain to the specimen platform. Too much glue can easily bleed to the edge of the platform and cause it to stick to the outer tube, preventing advancement of the block for cutting.

10. Cut coronal cortical slices at the preferred thickness. We typically use 300-μm thick slices [Figs. 3(e) and 3(f)].

**Tip**: We suggest discarding the first two or three slices before collecting, as these will often not be cut evenly.

**Tip**: The slice thickness may need to be adjusted for other brain regions. For example, light penetration for imaging studies will be low in heavily myelinated regions and thinner slices may be preferred.

**Tip**: The Compresstome cuts slices faster. This means gases and solutes provided in the slicing solution to maintain tissue health can diffuse into the tissue better and therefore slices will be healthier for longer. The Compresstome also does not use vibration during cutting, which, in our hands, keeps the tissue close to the surface of the slice healthier compared with the Vibratome. This is highly beneficial for NVC assays. Finding healthy blood vessels in slices can be a tough task and having a larger volume of healthy tissue, especially closer to the surface where light penetration, and therefore imaging, is best, is particularly helpful. Note, however, that one should not attempt NVC assay too close to the surface as it is important for the neuronal, astrocytic, and vascular architecture to be relatively intact.

11. Use a 7 mL Pasteur pipette with the tip cut off (to accommodate slice size) to transfer slices onto the submerged nylon mesh in the warmed slice recovery chamber containing slicing solution at 34°C. Make sure the slice recovery chamber is continuously, but mildly, bubbled. Slices are maintained in the warm bath for ~20 min.

**Tip**: If the rate of bubbling is too vigorous, large bubbles are injected into the solution, which attach to the underside of the nylon mesh while rising to the surface. This will compromise tissue health as the mesh regions with bubbles are regions with no salts and nutrients. Therefore, it is imperative that all bubbles stuck to the mesh are removed, and the bubbling is reduced to a mild level prior to transferring the slices. Bubbles stuck to the mesh can be easily dislodged by gently tugging the mesh with a pair of thin but blunt forceps or using a pipette to suck them out. Periodically check the mesh for bubbles throughout.

12. After 20 min, transfer slices from the warm recovery chamber to the storage chamber containing continuously bubbled storage solution at room temperature. Allow the slices to recover for 15 to 30 min before experiments.

**Tip**: As outlined in step 11, make sure there are no bubbles in the chamber and the bubbling is mild.

13. Slices in the continuously bubbled storage chamber can be used for up to 5 to 6 h after dissection, although the number of healthy vessels per slice will decline over time, especially in older animals.

### 12.2 Imaging Blood Vessels

**Fig. 4:**
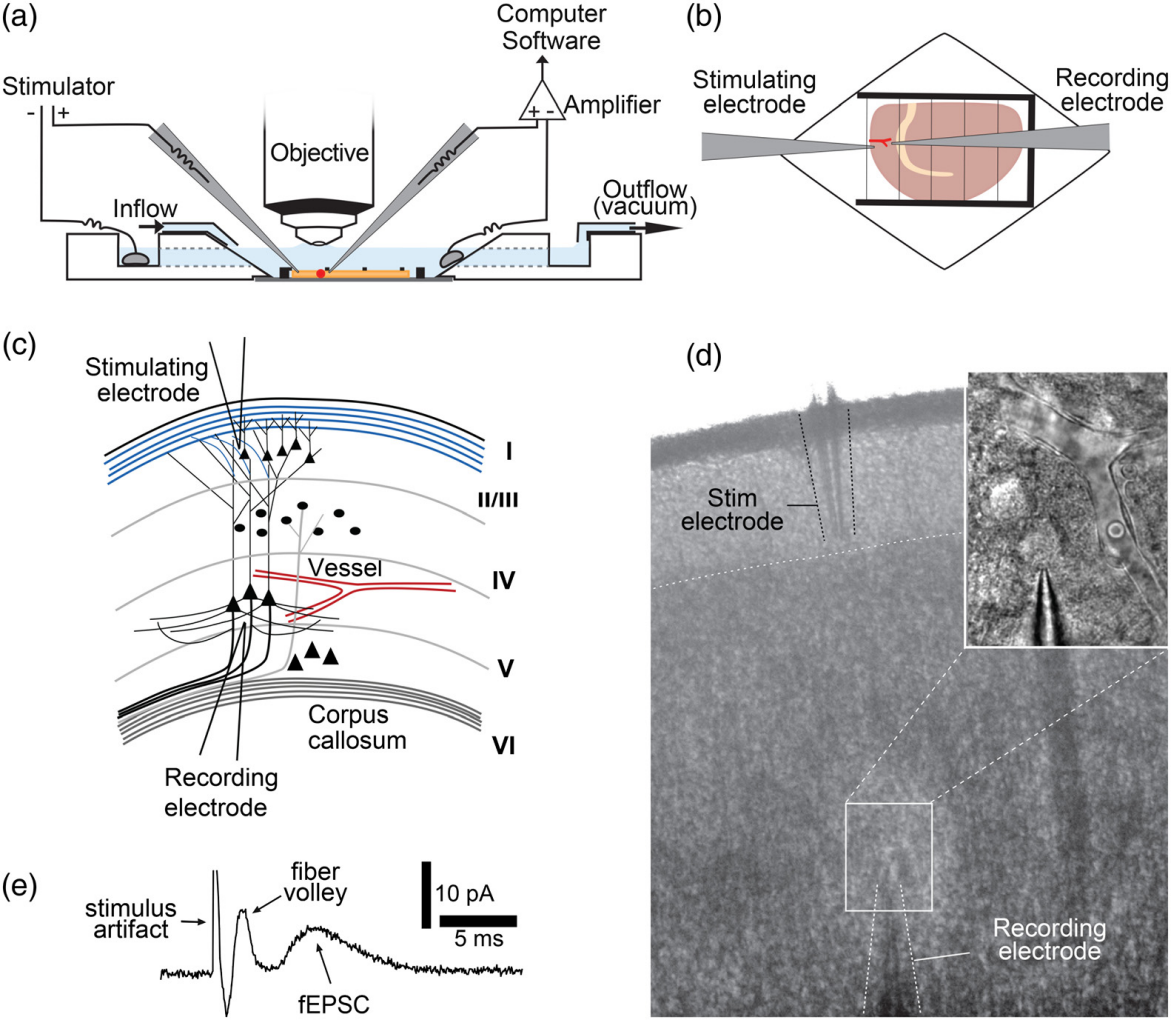
Placement of electrodes for cortical stimulation and field potential recording. (a) Schematic illustration of the cortical slice preparation for simultaneous imaging of blood vessels and electrophysiology. The slice is depicted in the imaging chamber as an orange slab, with a red dot representing the vessel of interest focused under the objective. (b) The slice is secured with a harp. The recording electrode is placed adjacent to the vessel of interest and the stimulating electrode is placed in layer I/II close to the pial surface. (c), (d). Detailed schematic illustration (c) and low magnification image (d) of a slice demonstrating appropriate electrode placement. Inset on upper right in (d) shows the recording electrode relative to the capillary of interest. (e) An example recording demonstrating the stimulation-evoked fiber volley and fEPSPs (measured as the current needed to hold the electrode at 0 mV; labeled fEPSCs), representing pre- and postsynaptic activity of neurons, respectively. (c)–(e) Adapted from Ref. 15.

14. At the time of experiment, transfer a slice from the storage chamber to the imaging chamber under the microscope. Secure the slice in the imaging chamber using a slice anchor or harp [Figs. 4(a) and 4(b)].

**Tip**: We recommend placing the cortical slice with the dorsal side (pial surface) facing the stimulating electrode, which will make electrode placement easier [Fig. 4(b)].

**Tip**: Temporarily halting the vacuum by kinking the vacuum tube can help stabilize the slice in the desired orientation before anchoring in place. Remember to restart the vacuum line to prevent flooding.

**Tip:** Stable and continuous slice perfusion is very important for maintaining $O_{2}$ levels and healthy tissue. Solutions should be continuously bubbled with 20% $O_{2}$. As much as possible, we recommend using tubing that is gas impermeable to perfuse the slice chamber. Further, reducing the length of the tubing from the aCSF container to the slice to be as short as possible will minimize gases escaping through the tubing. Solution should be flowing at a rate of 2 to 3  mL/min to ensure oxygen and nutrient delivery.

15. Find the brain ROI (we will focus on the cortex here) under lower magnification (4×). Switch to a higher magnification water-immersion objective (40× or 60×) to visualize cells and vessels better. Healthy neural tissue is characterized by readily identifiable neuronal cell bodies.

**Tip**: The brain has a beautiful abundance of cell types that are visible under the microscope. Taking the time to observe and study slices in detail will help you evaluate slice health as well as recognize different cell types (e.g., telling apart VSMCs and pericytes). Avoid areas where swollen (or blebby) neuronal processes, shrunken cells, or bulbous fragments of dead cells are visible.

16. Scan the slice (either through eye-pieces or with camera, depending on experimenter comfort) for a healthy vessel of the desired type (arteriole or capillary). Healthy vessels are characterized by a clearly visible lumen. They may or may not have red blood cells. Do not use vessels that are collapsed or have swollen endothelial tube (see Fig. 1, for examples, of healthy vessels; Ref. 11 also catalogs some examples of healthy and unhealthy vessels). We recommend scanning the slice in a systematic manner every time (e.g., dorsal to ventral, medial to lateral) to avoid confusion regarding areas that have and have not been examined and increasing efficiency. Using the same system to scan each slice makes the search faster and valuable time is saved.

**Tip**: All arterioles in the cortex branch off pial arteries and penetrate the brain perpendicular to the pial surface. Therefore, searching for arterioles can be streamlined by scanning the cortical layers closest to the pial surface, then following them into deeper cortical layers. Capillaries can be trickier to find, especially in very young animals as the capillary network is still developing until ∼P15-P25.^37^ If you cannot locate capillaries, search by following a pial arteriole into deeper layers, as there are usually capillaries that branch out.

**Tip**: Spend time looking at and identifying different blood vessels, as it can take some practice to confidently distinguish between types (e.g., capillary versus small arteriole). See Sec. 3 (Fig. 1) (consult Ref. 11 for further tips on identifying vessel types).

**Tip**: Using infrared DIC (IR-DIC) optics will allow imaging of vessels deeper in the slice.

17. For NVC assays, imaging the vessel will occur alongside electrophysiological recordings. Therefore, it is necessary to consider the position of the vessel relative to its surroundings and make sure there is enough space for recording and stimulating electrode (e.g., they are not blocked by harp strings, or the vessel is not too close to the pial surface, preventing proper electrode placement).

### 12.3 Recording Field Potentials

18. Prepare glass recording electrodes by pulling thick borosilicate glass capillaries to create ∼3 to 5  MΩ resistance tip size using a micropipette puller. Prepare glass stimulating electrodes by pulling thin borosilicate capillaries and breaking the tip gently to enlarge it (∼15 to 25  μm).

**Tip**: The stimulating electrode tip can be widened by gently touching it to a piece of tissue paper.

**Tip**: Different types of electrodes (e.g., double barreled or concentric electrodes) may be employed to stimulate neural activity. The choice of electrode may help achieve finer spatial selectivity for stimulation. However, for the cortex, activating a larger area is beneficial to ensure the ROI is activated, and we have had best success with simple glass electrodes.

19. Once the blood vessel of interest is identified, the next step is to place electrodes in the tissue. Return to lower magnification, insert aCSF-filled recording and stimulating electrodes into the manipulators. Place reference electrodes into the bath. Center both electrodes under the field of view as close to each other as possible without touching or breaking the tips and slowly lower both electrodes to just above (∼50 to 100  μm above) the slice surface (approximately the z-axis level at which the harp strings are in focus).
20. Switch to higher magnification (40× or 60×) cautiously. Care should be taken during this step so as to not knock aside the electrodes or break the electrode tips. Depending on the alignment of the microscope, you should be able to locate both electrodes under high magnification easily. Manipulate the electrodes in place just above the slice in the region containing the blood vessel of interest using the focus knob and camera live image.

**Tip**: Verify again that electrode tips are of appropriate size. The recording electrode tip should appear no more than ∼2 to 3  μm wide (less than a third of the width of a red blood cell). Although the size of the stimulating electrode cannot be exactly controlled, it should be ∼15 to 25  μm wide.

21. Using the fine setting on your manipulator, carefully insert the recording electrode into the tissue within 10 to 30  μm of the blood vessel and approximately the same z-plane [Figs. 4(c) and 4(d)].

**Tip**: Move the electrode into position in the tissue by manipulating it in “diagonal” setting as much as possible rather than lowering it straight down. This will ensure that the tissue directly above and around the electrode are as intact as possible to obtain more robust field activity and minimize crushing or collapsing the vessel.

**Tip**: Check the electrode drift in your imaging rig. If they are drifting too much, this might push or pull the tissue around the vessel during imaging, which may artifactually change the vessel diameter. Calibrate and maintain the manipulator to ensure minimal drift.

**Fig. 5:**
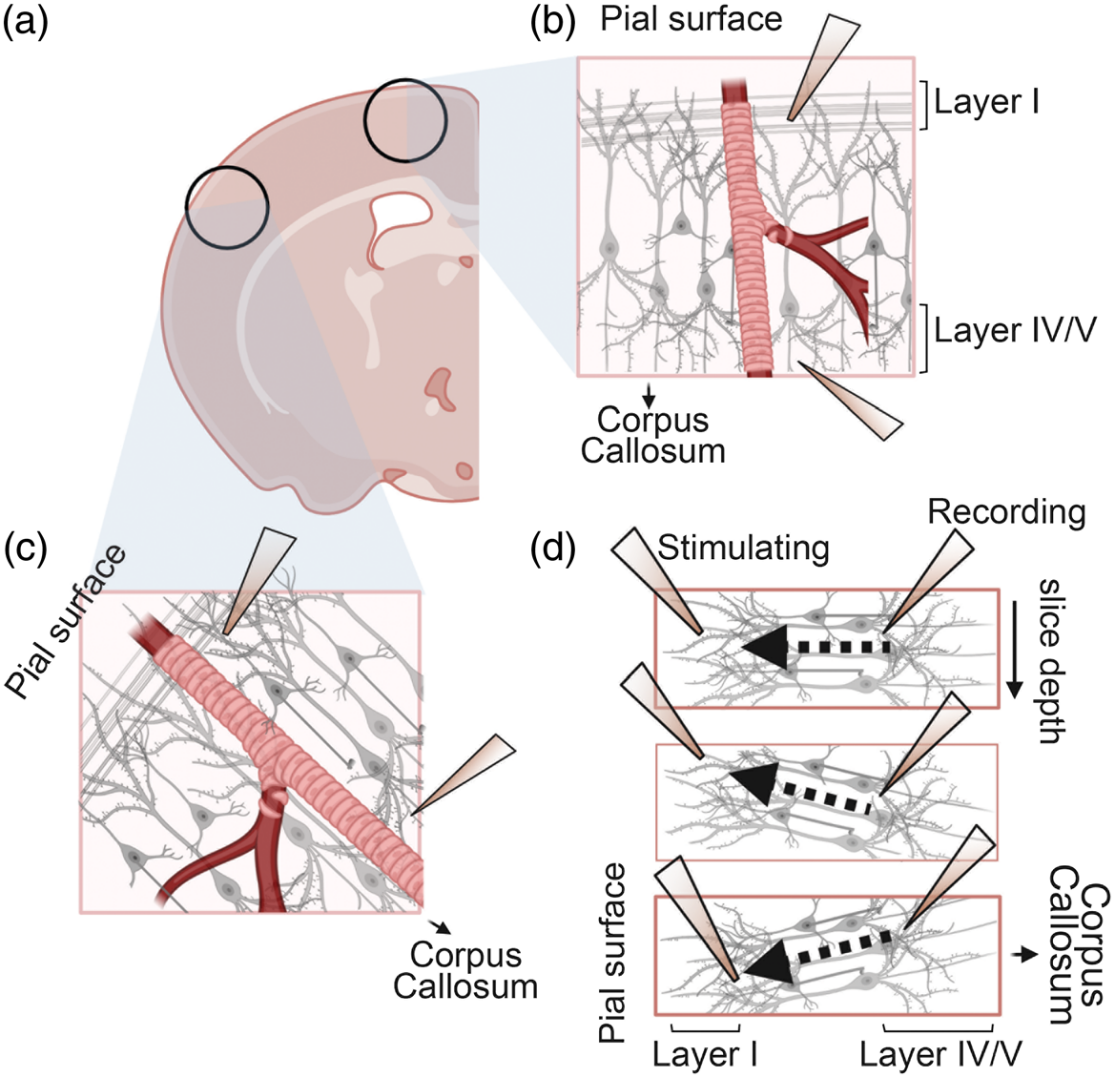
Placement of electrodes depends on direction of dendritic projections. (a)–(c). In a coronal slice, the direction of dendrites of neurons in deeper cortical layers is always perpendicularly toward the pial surface, but the relative direction under imaging optics will change depending on the location of the ROI. Dendrites of neurons in the ROI may be (b) extending straight or (c) diagonally depending on slice orientation. (d) The dendritic processes will also not always be in the same plane as the slice (top panel) but may be angled toward the top surface (middle panel), or deeper into the tissue (toward the bottom surface; bottom panel). The direction of dendrites in all three dimensions must be considered when placing the stimulating electrode to successfully activate the ROI. Created with BioRender.com.

22. Focus up and down to visualize the direction and angle (relative to the slice plane) of the dendrites of neurons surrounding the vessel. Using the fine setting on your manipulator, insert the stimulating electrode diagonally into layer I/II in the direction that the dendrites are pointing (toward the pial surface), ∼500  μm away from the recording electrode [Figs. 5(a)–5(c)]. The depth at which the stimulating electrode is placed will depend on the angle of the dendrites relative to the slice plane [Fig. 5(d)].

**Tip**: Take care to move the electrode into the tissue diagonally to minimize damage to tissue of interest.

**Tip**: Deciding where to place the stimulating electrode is a crucial step and depends on the direction of dendrites in all three dimensions. Visualize dendrites of individual neurons as well as the orientation of bundles of nearby dendrites to determine their direction in the x- and y-planes. If long segments of dendrites can be readily seen, this suggests the dendrites are in the same z-plane within the slice and the stimulating electrode can be placed at the same depth as the recording electrode. If only short dendritic processes are visible, they are either angling deeper or shallower relative to the slice plane. Spend some time identifying which direction (shallower or deeper) the dendrites are heading by focusing up and down, then place the stimulating electrode accordingly (see Sec. 4 for more discussion).

**Tip**: As the stimulating electrode tip is large, it will tear and damage the tissue as it moves through the slice. When in doubt, it is best to start shallower and further away, then adjust by slowly moving deeper and closer to the region with the recording electrode/vessel as necessary. It is worthwhile practicing electrode placement for reliable field potentials in different regions of the cortex before commencing experiments.

**Tip**: The harp should be flat and level such that the slice sits flat in a stable manner under the strings. If the harp does not hold the slice firmly, the slice can drift with even slight changes in perfusion flow and the electrodes may be jostled out of place or focus may be lost during imaging. Further, the harp strings should be spaced close enough to hold the slice stable, but far enough that they do not impede appropriate electrode placement or obscure large segments of the slice.

23. Once both electrodes are in place, deliver a single test pulse. Proper electrode placement will result in the recording electrode detecting the stimulation artifact, the fiber volley, and the field postsynaptic activity [Fig. 4(e)].

**Fig. 6:**
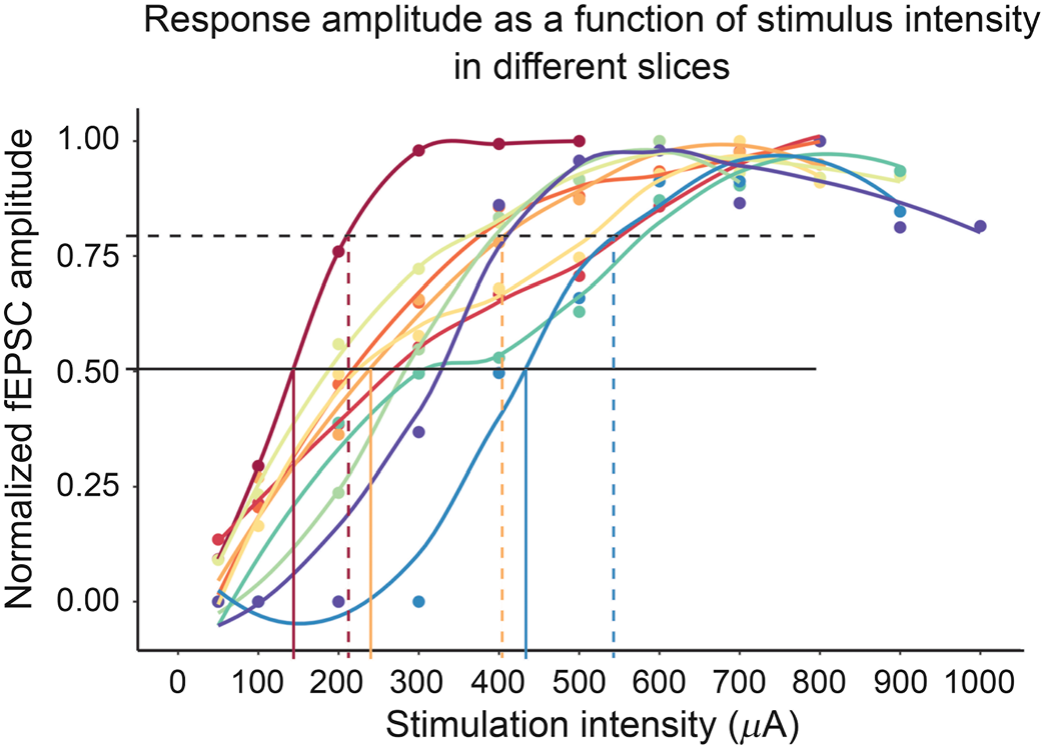
Stimulation intensity required to induce maximal field activity in different slices. Ten different slices were stimulated with 50, 100, 200, 300, 400, 500, 600, 700, 800, 900, 1000  μA stimulus pulses, until a maximum peak was reached. Maximum intensity was determined as two stimulation intensities resulting in a similar field potential peak amplitude. Stimulation protocol was a train of 5 pulses at 1 Hz at each intensity with ∼10  s of rest between trains. The average peak amplitude was plotted at each stimulation intensity. The scatter plot for each slice was fit with a nonparametric loess regression in R. Solid black line depicts the 50% and dashed black line depicts the 80% peak intensity. Note that the stimulation intensity required to reach 50% peak intensity (solid black line) or 80% peak intensity (dashed black line) differs in each slice. As examples, three sets of vertical lines are shown in red, orange, and teal, depicting the stimulus intensity required to reach 50% (solid lines) or 80% (dashed lines) peak for three slices.

**Tip**: The stimulus intensity to be used may be predetermined based on preliminary testing. However, to achieve more consistent levels of synaptic activation across experiments, we recommend testing a series of stimulus pulses at increasing intensities in each slice at this stage until the maximum slope or amplitude is achieved, and then use a preset cutoff of this stimulus for the actual experiment (e.g., intensity required to get 50% or 80% activation, as preferred). The stimulus intensity to reach maximum response can vary quite significantly between preparations (Fig. 6). Note that these test pulses should be separated by several seconds so as not to affect the blood vessel. Once the electrodes are in place, it only takes about 1 to 2 min to complete this step, so adding it will not unduly lengthen the experiment duration (thus concerns about slice health are avoided).

24. The preparation is now fully set up to perform the NVC assay. Using the imaging software, begin capturing a time series. We capture images every 5 s, which serves to adequately capture changes in the blood vessel for slice NVC experiments.

**Tip**: Imaging at a faster rate may allow one to capture responses potentially occurring at faster timescales, but this will of course also increase the file size, which can add up quickly. Make sure you have abundant free disk space prior to commencing experiments.

25. After imaging the vessel in control aCSF for 3 to 5 min to record the baseline diameter, switch the perfusion solution to aCSF containing U46619 (or the preconstrictor of choice). Incubation in 200 nM U46619 causes a modest level of vessel constriction (∼15% to 20% on average), although the extent of the constriction is variable and can be dependent on other factors, such as age, type of vessel, and brain region. Keep track of exact time points when conditions are changed. Monitor the vessel carefully to see if you can detect the constriction. This is sometimes salient, but often subtle. Continue imaging in U46619 for 5 min at a minimum.

**Tip**: If the imaging plane is deeper in the slice, the bath-applied drugs will take longer to diffuse through the tissue into the ROI and thus be able to induce vessel tone. If you notice the vessel is just starting to constrict toward the end of 5 min, give it another few minutes to stabilize.

**Tip:** It is important to continuously monitor that the vessel remains in focus. Shifts of the tissue in the z-plane or movements in the slice, especially those caused by the slice-wide vasoconstriction induced by U46619 (or other constrictor of choice) can make the vessel move out of the focal plane. Small adjustments in focus may be necessary to keep the vessel in focus. **Important note:** Use nonvessel landmarks within the tissue to monitor the focal plane. Vessel movements could be due to focal plane changes or vasoactivity; therefore, the vessels themselves are not reliable for monitoring focal plane. Instead, this is easily assessed by finding a set of cells or subcellular structures in image that will serve as a proxy for correct focal plane.

**Tip**: If nothing is detected by the recording electrode, first make sure the stimulator is on and the wires are connected properly to the anode and cathode ends. Make sure the reference electrodes are immersed in the bath and there are no breaks in the circuit. If the stimulus artifact is obvious but no activity is detected, reevaluate the direction and angle of the dendrites and reposition the stimulating electrode. If the fiber volley is present but field postsynaptic activity (fEPSP or fEPSC, depending on the recording settings) is absent, again try moving the stimulating electrode and, to a lesser extent, the recording electrode to test if it can be improved. However, presence of fiber volley without synaptic activity may indicate the tissue is not healthy, so it may be best to start anew with a fresh slice.

### 12.4 Perform the NVC Assay

26. Optional step: If using other drugs to test their effect on NVC (e.g., inhibiting a putative vasoactive pathway), switch the perfusion solution to aCSF containing the drug of interest in addition to U46619. U46619 must continue to be present at the same concentration to maintain constant basal tone. Continue imaging in the presence of the drug for at least an additional 5 min to give it time to exert its effect (or as long as necessary given the time-course of the drug’s action).
27. After the vessel has achieved tone and stabilized in the preconstrictor or the drug of interest, stimulate electrical activity with a predefined protocol while capturing the field activity using the relevant software. We deliver 200−300  μA pulses for 3 s at 20 Hz to stimulate the tissue and record the resultant activity using the Clampex module of pClamp. Continue imaging for another 5 min to capture the full response of the vessel to the stimulated activity.

**Tip**: Neurons in different brain regions have different properties. Determine the appropriate stimulation strength and frequency based on the brain region and other relevant conditions (e.g., age). This should be done prior to all experiments and kept consistent throughout the individual study.

### 12.5 Analysis

28. Criteria for analysis should be predetermined and adhered to consistently. We employ three primary criteria for pursuing analysis of NVC assays: (a) The vessel must constrict to U46619 (or the preconstricting agent of choice), thus providing tone to the *ex vivo* vessel. As smooth muscle or pericyte contraction is the active component of vascular diameter regulation (and relaxation, leading to dilation, is essentially inhibition or relief from contraction), this also ensures that the vessel of interest is healthy and responsive. (b) Field activity of a sufficient amplitude must be generated following the stimulation during the assay, ensuring that neurons in the region were indeed activated during the experiment. (c) The vessel of interest must stay in focus throughout the experiment.
29. Using an image analysis software such as MetaMorph or ImageJ (FIJI), measure the diameter of the vessel in each image of the time series captured. Quantify the change in diameter to each change in condition (e.g., U46619 and drug application) and following stimulation. We quantify responses as a percent change in diameter normalized to the baseline diameter before the application of any drug (including U46619). This may slightly underestimate the response (as normalizing to a larger diameter, prior to preconstriction, will produce a smaller % response). However, we prefer this to overestimating the response (which may be the case if normalized to the diameter after preconstriction). The simplest NVC analysis will include the vessel diameter at baseline, the constriction evoked by U46619, and the peak response (dilation or constriction) following neural stimulation.

**Tip**: Keep precise notes on the time and image frame when the drugs or stimulation were delivered. The onset of vessel response can sometimes be very fast and other times it can be slower. To ensure that vessel response being quantified is actually evoked by the stimulation-induced neural activity, use a predetermined time window within which the response must begin and confirm with parallel experiments in which synaptic activity is inhibited.

**Tip**: Regions of the vessel that produce the strongest constriction to U46619 are likely the healthiest segments, or, in the case of capillaries, segments that harbor contractile pericyte processes. Quantify the vessel’s response to stimulation in the same regions. Note that vessel constriction (contraction of the VSMC or pericyte) is the active component and dilation (or relaxation of the VSMC or pericyte) is essentially an inhibition of, or relief from, contraction.

30. Analyze the field activity recordings using an appropriate software (e.g., Clampfit module of pClamp). You may choose to quantify several components of this response, such as the amplitude, rising slope, and area under the curve, as relevant for the experimental context.

## 13 Anticipated Results

This protocol provides a step-by-step guide to performing NVC assays in *ex vivo* acute slices of rodent brains. It contains a variety of technical considerations such as dissection methods and speed, brain slice quality, blood vessel types, and electrical stimulation of neural activity. These experiments are ideal for studying specific signaling mechanisms contributing to NVC. Identified mechanisms can then be translated to *in vivo* studies to interrogate functional hyperemia.

An experimenter who is comfortable with basic imaging and/or electrophysiological skills should be able to replicate these assays with relative ease. For beginners, mastering all the aspects necessary to successfully perform NVC experiments may take several months. Successful experiments require all cell types in the neurovascular unit to be functional and the intercellular signaling mechanisms intact. Therefore, slice quality is a critical factor and can be improved with increased speed and accuracy of dissection. The overall structural integrity of the tissue and vessels is a good first-pass assessment of tissue health. Neuronal health is more thoroughly indicated by the robustness of the field activity recordings, whereas vessel health is indicated by their response to the preconstricting agent. If either of these are absent, the region is not healthy enough to carry out the assay and must be abandoned. This is not uncommon; therefore, it is useful to create as many slices as possible from each animal to ensure several successful experiments. We routinely collect 10 to 14 slices and perform 2 to 6 successful NVC assays per day.

**Fig. 7:**
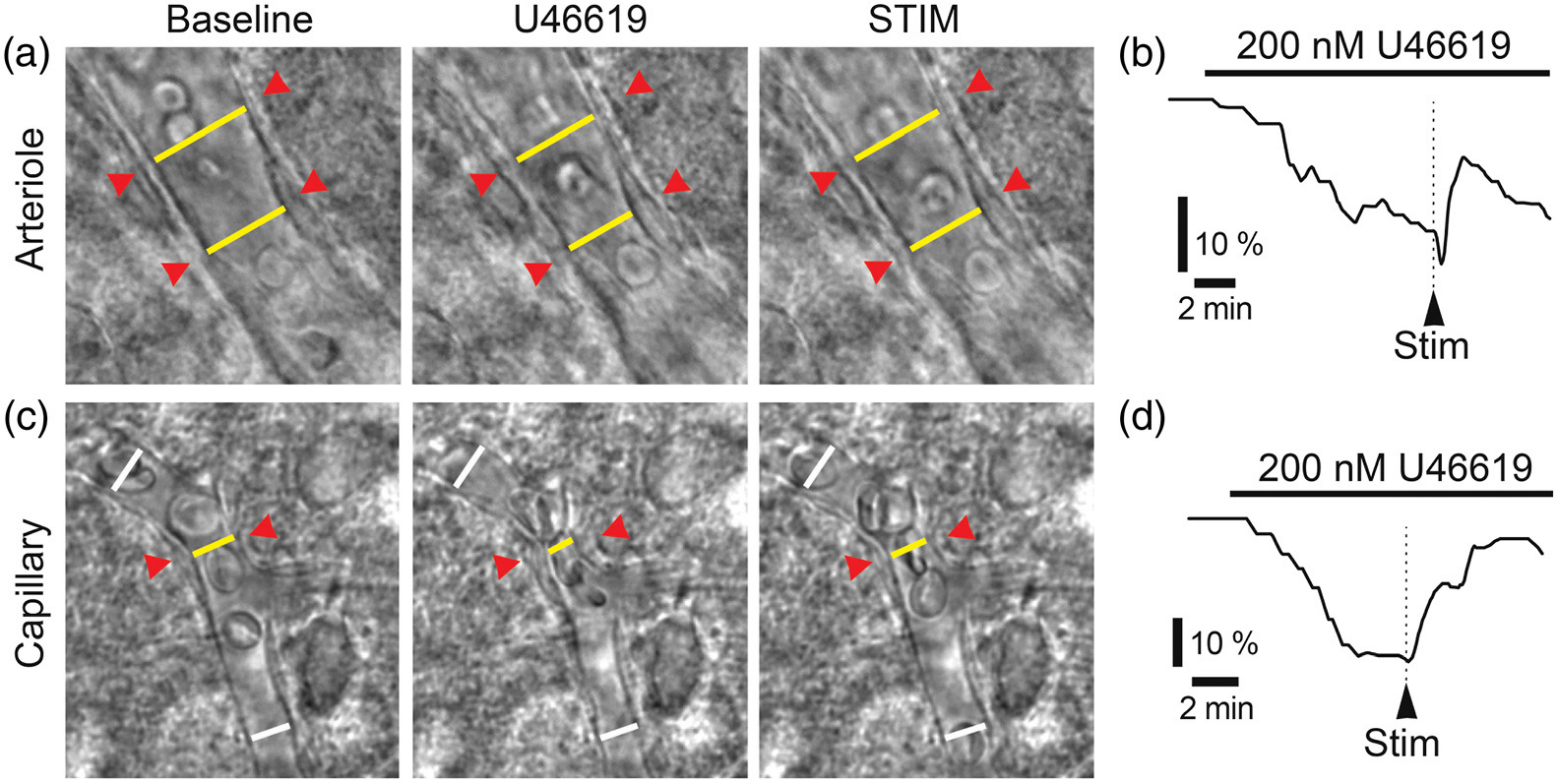
Arteriole and capillary responses to cortical stimulation. (a) An example arteriole at baseline, following U46619-induced constriction, and after stimulation (STIM). (b) The diameter of the vessel shown in (a) plotted over time, showing the change in arteriole diameter in response to U46619 and neural stimulation. A video of the same arteriole is available online (Video 1, MOV, 1.61 MB [URL: https://doi.org/10.1117/1.NPh.9.3.031913.1]). U46619 and neuronal stimulation (red circle) were applied at the frames indicated. Video is played at 80× speed. (c) An example capillary at baseline, after U46619-induced constriction, and after stimulation and (d) the corresponding diameter trace demonstrating the response of the capillary to each manipulation. A video of the same capillary is available online (Video 2, MOV, 1.23 MB [URL: https://doi.org/10.1117/1.NPh.9.3.031913.2]). U46619 and neuronal stimulation (red circle) were applied at the frames indicated. Video is played at 50× speed. Yellow lines flanked by red triangles in (a) and (c) indicate the diameter of the vessels in each condition. The arteriole response plotted in (b) was averaged from measurements from both marked locations. Two regions marked with white lines in (c) depict regions of the capillary that do not change in diameter. Note that this is typical of capillaries, as pericytes are spatially separated and their processes do not contiguously cover the vessel.

Example experiments on an arteriole and a capillary are shown in Fig. 7 and Videos 1 and 2. In both experiments, 200 nM U46619 was used as the preconstrictor. Note the time-locked dilation that follows immediately after neuronal stimulation. By performing similar experiments in the present of different agonists and/or antagonists, one can start narrowing the signaling pathways that are involved in NVC under different conditions (vessel type, disease, etc.; see Refs. 11 and 14, for examples) or in different regions of the brain.

Following the NVC assay, blood vessel diameter is measured in all images of the time series, and the resulting data are further processed to quantify changes in vessel diameter in response to different manipulations (e.g., drug application or neural stimulation). During analysis, it can sometimes be difficult to distinguish changes in focus from an actual response of the blood vessel. Shifts in the X-Y plane can also make analysis challenging. The quality of the harp is an essential factor in holding the slice stable in both the X-Y plane and maintaining stable focus, hence preventing such issues (see Ref. 11 for more detailed instructions). *Post-hoc* image alignment using registration tools available in many image analysis software (e.g., the StackReg Plugin in ImageJ/FIJI) may also help with some X-Y drift. Ensure that the diameter measurements are made at the same location of the vessel on each image of the time series using nearby landmarks such as neuronal cell bodies.

## 14 Benefits and Limitations of Brain Slice NVC Experiment

### 14.1 Benefits

Working with brain slices is more accessible to a wide array of scientists worldwide, as they require fewer resources and are more economically affordable. As experiments are performed in tissue harvested post-mortem, it is also considerably less invasive on live animals compared to *in vivo* experiments. Brain slices provide an easily manipulatable system for pharmacology studies, as drugs can be easily introduced globally into the bath or locally via puffing, whereas concerns regarding blood–brain permeability, metabolism (e.g., in the liver), or adverse effects on other organ systems are effectively sidestepped. This allows researchers to interrogate many pathways in a relatively short period of time, saving valuable time and resources. The brain slice preparation also allows one to study the effect of vasoactive signals generated by neural activity on particular vascular segments. As the slicing procedure disconnects the vascular tree and depressurizes the blood vessels, neuronal activity can engage active, local responses only, whereas passive effects from up- or downstream vessels are avoided. Being able to stimulate neuronal activity and measure the subsequent vessel response is another important feature, because such stimulation causes neurotransmitter release and postsynaptic as well as glial activation. This method is more relevant to *in vivo* NVC than global application of neurotransmitters (such as glutamate) or vasoactive agonists.^11^^,^^12^^,^^14^ Therefore, while the brain slice is a simplified reduced system, it can be quite efficient in investigating mechanisms that drive NVC.

### 14.2 Limitations

Although this technique is useful for studying the relationship and specific signaling pathways between neuronal, glial, and vascular components of the brain, there are some major methodological challenges that one needs to keep in mind. In an acute slice preparation, the brain is removed from the body and thinly sliced, then maintained in buffered solutions matched to the brain’s extracellular ionic milieu for a period of hours. This is an obviously traumatic procedure on the tissue that may change neurophysiology in numerous subtle or overt ways; thus, phenomena observed in slice preparations may not always reflect processes *in vivo*. In addition, one should carefully construct the stimulation protocol and place the electrodes such that neuronal activity can be elicited without directly affecting the vasculature. Direct electrical stimulation of blood vessels will depolarize mural cells, especially VSMCs, and therefore trigger a constriction. Such direct effects can often be recognized by the almost nonexistent latency between electrical stimulation and constriction and must be avoided to observe accurate neurovascular responses. Further, due to the lack of intraluminal pressure and flow, mechanosensitive mechanisms that may help re-establish tone of vessel *in vivo* are not active in slices and recovery of tone is often very slow; hence, this technique may not be useful in studying the recovery phase of the vasoactive responses. Lastly, the increase in CBF evoked by NVC *in vivo* is shaped by an integration of signals from neurons and glia, independent responses of different mural cells along the cerebrovascular tree, conducted propagation along the endothelium, as well as passive changes forced by local as well as systemic flow/pressure changes. This complex interaction of the cerebrovascular system cannot be replicated in slices. Therefore, brain slice NVC assays can serve as a fertile method to narrow the mechanisms involved in NVC but these findings must always be verified *in vivo*.

## 15 Conclusions

The NVC response is imperative for healthy brain function, but changes during development^6^^,^^38^ and in the context of several neurological diseases.^13^^,^^39^^–^^42^ Although these conditions must be understood, ultimately, in the *in vivo* context, drug delivery to the brain for refined *in vivo* pharmacology studies can be challenging. Furthermore, it is difficult to distinguish *in vivo* the active, local response of different vascular segments from passive effects due to changes in flow or pressure within that vascular tree. For instance, dilation of capillaries observed *in vivo* have been suggested to occur due to passive stretch in response to the increase in flow effected by the dilation of upstream arterioles, or even to red blood cells “pushing” open the capillaries as they move through. No question that these factors also come into play *in vivo*, but the fact that pericyte-mediated capillary dilations are observed in brain slices, where arteriole and capillary segments are disconnected from each other in addition to the absence of lumenal flow, suggests that capillary diameter can also be locally and actively regulated (supporting findings that capillaries respond before arterioles *in vivo*^14^^,^^24^). Therefore, using acute brain slices for *ex vivo* NVC assays as described herein may assist in the discovery of specific mechanisms that regulate or impair NVC at specific levels of the vascular tree in different contexts of development or disease. Such studies should prove a valuable complement to *in vivo* studies to enhance our understanding of how the brain’s energy supply is maintained.
